# An EZ Bayesian hierarchical drift diffusion model for response time and accuracy

**DOI:** 10.3758/s13423-025-02729-y

**Published:** 2025-07-25

**Authors:** Adriana F. Chávez De la Peña, Joachim Vandekerckhove

**Affiliations:** 1 Department of Cognitive Sciences, University of California, Irvine, CA USA; 2 Department of Statistics, University of California, Irvine, CA USA

**Keywords:** Hierarchical Bayesian, EZ diffusion, Cognitive psychometrics, Indirect inference

## Abstract

The EZ-diffusion model is a simplification of the popular drift diffusion model of choice response times that allows researchers to calculate diffusion model parameters directly from data with no need for expensive computations. The EZ-diffusion model is based on a system of equations in which the diffusion model’s drift rate, boundary separation, and nondecision time parameters are jointly used to predict three summary statistics (the accuracy rate and the mean and variance of the correct response times). These equations can then be inverted to obtain estimators for the three parameters from these summary statistics. Here, we describe a probabilistic formulation of the EZ-diffusion model that can serve as a hyper-efficient proxy model to the drift diffusion model. The new formulation is based on sampling distributions of summary statistics and consists only of normal and binomial distributions. It can easily be implemented in any probabilistic programming language. We demonstrate the validity of the proxy model through extensive simulation studies and provide multiple examples (via https://osf.io/bzkpn/), including an implementation in JASP. We conclude that, although the recovery of some parameters with the proxy model is biased, the recovery of regression parameters is good, making the method useful for cognitive psychometrics (i.e., explanatory cognitive modeling). Casting the EZ-diffusion model in the broad family of Bayesian generative models allows us to benefit from mature implementations, practical workflows, and powerful extensions that are not possible without a probabilistic implementation and not feasible with the regular drift diffusion model. Code and example applications are provided via https://osf.io/bzkpn/.

*Cognitive psychometrics* is a relatively new, specialized discipline in which advances in cognitive modeling are applied in psychological measurement (Batchelder, 2016). The advance of cognitive psychometrics reflects a broader trend towards the use of idealized models in the philosophy of measurement (Tal, 2020).

The relationship between cognitive science and cognitive psychometrics is by nature symbiotic: As cognitive scientists develop ever-more sophisticated models of cognition and behavior, so are psychometricians empowered to use those models as measurement tools. The goal of cognitive psychometrics is to construct an algebra of data, so that complex and nonlinear data patterns can be expressed as simple composites of interpretable units. An example success story of this research program is the drift diffusion model (DDM) for two-choice response times (RTs). The model was developed, gradually, by Stone (1960) and Link (1975). It was then made popular through extensive work by Ratcliff (1978), who showed that diffusion model parameters behave like their namesakes in a wide variety of contexts (Wagenmakers, 2009; see also Voss, Rothermund, & Voss, 2004; but see Lerche & Voss, 2018, and Rafiei & Rahnev, 2021, for violations of selective influence). After that, the model was developed into a measurement tool (Vandekerckhove, Verheyen, & Tuerlinckx, 2010; Vandekerckhove, Tuerlinckx, & Lee, 2011), and many interesting applications have resulted (see, e.g., Ratcliff, Smith, Brown, & McKoon, 2016). Taken together, this is a cognitive psychometric research program, intended to quantify patterns of data that are indicative of underlying latent features that vary between individuals and other empirical units. When quantified, we may then seek to *explain* the observed variability (De Boeck & Wilson, 2004; Vandekerckhove, 2014).

Theoretical progress in cognitive psychometrics is achiev-ed through the development of cognitive models to describe the latent processes that generate data. Ideally, the models specify such processes with parameters that carry psychological meaning and that can be interpreted in the context of the data collection, while formalizing only those substantive assumptions about cognition to which the researcher is willing to commit.

Some of these models, however, are somewhat involved complex and require significant modeling expertise to implement. The DDM in particular became popular for its ability to account for data patterns that are known to be elicited by specific aspects of experimental task designs, but its implementation is computationally complex, erecting a barrier to applications. In response to these limitations, Wagenmakers, van der Maas, and Grasman (2007) developed an “EZ” version of the DDM that allows the user to estimate the model’s key parameters from summary statistics of observed choice RT data (i.e., the mean and variance of the correct RTs and the accuracy rate). The EZ method (hereafter, EZ-diff) has since been widely applied in the study of, among others, perceptual decision-making (Bitzer, Park, Blankenburg, & Kiebel, 2014; Mulder, van Maanen, & Forstmann, 2014), of working memory and intelligence (Schmiedek, Oberauer, Wilhelm, Süß, & Wittmann, 2007), of self-regulation (Enkavi et al., 2019).

The Bayesian implementation of cognitive process models is crucial to the cognitive psychometrician, as it facilitates their application as measurement models. Cognitive process models that are typically used to account for the underlying response processes at the individual level can be extended hierarchically to take into account between-subjects variability and capture individual differences (Lee, 2011; Vandekerckhove et al., 2011). Furthermore, models can be extended into cognitive latent variable models that distinguish across different levels of variation, or incorporate meta-regression structures that capture predictors associated with any of the parameters (Vandekerckhove, 2014).

To deal with complex models with intractable likelihoods, statisticians will sometimes adopt so-called ‘indirect inference’ approaches, in which a target likelihood is approximated by an auxiliary or proxy model (Jiang & Turnbull, 2004; Price, Drovandi, Lee, & Nott, 2017). For example, Wood ’s (2010) synthetic likelihood method models relevant summary statistics using a multivariate normal distribution.^1^

In this paper, we propose a similar approach to working with the DDM. We construct a proxy model from the sampling distributions of the EZ-diffsummary statistics. This simplified model can easily be implemented in specialized software as a Bayesian generative model, allowing for the implementation of hierarchical and explanatory meta-regression structures. We demonstrate the viability of this application through multiple examples developed in JASP and R (via https://osf.io/bzkpn/). First, we will review the DDM and EZ-diff. We will then introduce our proxy model, and then illustrate how it can be made into a proxy for a Bayesian hierarchical DDM.

## The drift diffusion model

The drift diffusion model (DDM) is a cognitive process model that describes binary choice RT data as the result of a stochastic sampling process. The core assumption is that decision-makers accumulate information about stimuli presented, starting the moment they are asked to make a binary judgment and ending once a decision boundary is met. One implication of the principle of accumulation of information (Laming, 1968) is that people make two decisions: when they are ready to respond and what to respond. These aspects are captured by the RT and choice data, respectively.

**Fig. 1:**
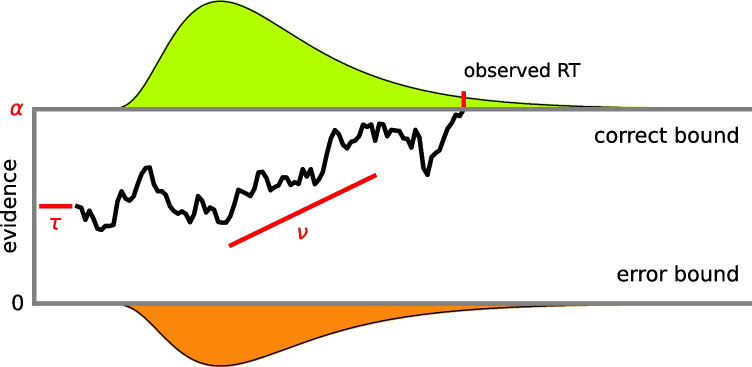
Evidence accumulation in a two-choice decision task. The *jagged line* represents the noisy process of evidence accumulation over time until the observed reaction time, when the evidence hits the ‘correct’ decision bound. The distributions are predicted RT distributions for correct decisions (*top*) and errors (*bottom*). The drift rate $\nu$ captures the participant’s evidence accumulation rate: their ability at the task. The boundary separation $\alpha$ captures the participant’s speed–accuracy trade-off: their caution at the task. The nondecision time $\tau$ captures the time spent on processes other than decision-making. Figure credit Vandekerckhove and Chávez De la Peña (2023)

The three key parameters of the DDM are illustrated in Fig. 1. The drift rate parameter $\nu$ (nu) indicates the average amount of evidence sampled per unit of time; the boundary separation parameter $\alpha$ (alpha) corresponds to the distance between the two response boundaries; and the non-decision time parameter $\tau$ (tau) accounts for the time required to encode and process the information presented by the stimuli. The parameters of the DDM capture relevant aspects of the psychological process that underlies decision-making. These aspects can be manipulated through experimental design: The drift rate is affected by the quality of the information conveyed by the stimuli and by individual differences in processing efficiency. The boundary separation captures the speed–accuracy trade-off imposed by task instructions and serves as an indicator of individual caution. The nondecision time is an indicator of the perceptual complexity of the stimulus (encoding time) and of the response modality (motor response time), and seems to be affected by stimulus complexity (Nunez, Gosai, Vandekerckhove, & Srinivasan, 2019) and participant age (Ratcliff, Thapar, & McKoon, 2001).

The DDM is frequently implemented as a cognitive process model to account for the underlying mechanisms that generate the collected data. In contrast, cognitive psychometricians use the DDM as a statistically tractable measurement tool that can be extended to explore latent variable structures, quantify individual differences, and estimate regression coefficients for covariates of interest (Rouder, Province, Morey, Gomez, & Heathcote, 2014).

In the present paper, we focus on the latter case: the implementation of the DDM as a measurement model to explain variability in the model’s parameters with exogenous predictors. The practical application of a process model as a measurement model will benefit from the use of a Bayesian hierarchical model (Dutilh et al., 2017; Rouder & Lu, 2005; Rouder et al., 2014; Rouder & Haaf, 2019; Schubert, Nunez, Hagemann, & Vandekerckhove, 2019; Vandekerckhove et al., 2011; Villarreal et al., 2024).

## Hierarchical cognitive models

Cognitive models are traditionally implemented to describe individual-level performance (e.g., Lee, Newell, & Vandekerckhove, 2014), with group performance being described using aggregate measures that assume low between-subject variability (Estes, 1956). This traditional approach implies a multi-step analysis that starts by estimating individual parameter values and then using summary measures for inference regarding average group performance and individual differences (e.g., Klauer, Voss, Schmitz, & Teige-Mocigemba, 2007). While this two-stage approach is common, it is important to remember that the parameters estimated in the first stage have uncertainty associated with them (either posterior variability or standard error of measurement), and for the purposes of statistical inference, this uncertainty needs to be propagated appropriately. Neglecting this uncertainty in the second stage leads to a risk of so-called *generated regressor bias* (Pagan, 1984; Boehm, Marsman, Matzke, & Wagenmakers, 2018).

Hierarchical cognitive models are extensions of cognitive models that model multiple sources of variability simultaneously (e.g., measurement error, variability between individuals, stimuli, experimental conditions, etc.; Coleman, 1964; Clark, 1973). In these models, parameter values across levels of variation are assumed to be sampled from parent distributions (Lee, 2011), such that variability in individual performance is taken into account when estimating population-level statistics (Lee & Webb, 2005; Rouder & Lu, 2005). In the context of nonlinear models of cognition, hierarchical modeling is especially critical because generated regressor bias may be asymptotic (i.e., the bias does not go to zero even with very large samples; Rouder & Lu, 2005).

While it is possible to implement hierarchical cognitive models under any statistical philosophy, it is often most convenient to make use of flexible Bayesian modeling strategies (Lee & Wagenmakers, 2013), and this is the default practice.

Going forward, we will use the following notational conventions. The choice and RT $\textbf{y}_{pi}$ of person *p* on trial *i* is modeled using a Wiener distribution with participant-specific drift rate $\nu _p$, boundary separation $\alpha _p$, and nondecision time $\tau _p$ (Eq. 1). The hierarchical model assumes that individual parameters $\nu _p$, $\alpha _p$, and $\tau _p$ are sampled from parent normal distributions with a mean and variance that describe the population (Eqs. 2, 3, and 4).

1 $$\begin{aligned} \textbf{y}_{pi}\sim &  \text{ Wiener }\left( \alpha _{p},\, \tau _{p},\, \nu _{p}\right) \end{aligned}$$

2 $$\begin{aligned} \nu _{p}\sim &  \text{ Normal }\left( \mu _\nu + \beta x_p,\, \sigma ^2_\nu \right) \end{aligned}$$

3 $$\begin{aligned} \alpha _{p}\sim &  \text{ Normal }\left( \mu _\alpha ,\, \sigma ^2_\alpha \right) \end{aligned}$$

4 $$\begin{aligned} \tau _{p}\sim &  \text{ Normal }\left( \mu _\tau ,\, \sigma ^2_\tau \right) \end{aligned}$$

Equation 2 includes by way of example a *metaregression structure* on the drift rate. Here, person-specific drift rates are modeled as samples from normal distributions that are shifted from a shared population mean by an individual predictor *x* (indexed with *p*) multiplied by a regression coefficient $\beta$. Similar regression structures can be applied to the boundary separation and nondecision time parameters,^2^ and nonlinear regressions may be implemented as well.

## The EZ-diffusion model

The “full” DDM accounts for empirical data patterns commonly observed in binary choice tasks through the implementation of trial-by-trial variability in the drift rate, starting point, and nondecision time (Ratcliff, 1978; Ratcliff & Rouder, 1998; Ratcliff & Tuerlinckx, 2002). However, this seven-parameter version of the DDM is significantly more complex and requires specific modeling expertise to be implemented (Vandekerckhove & Tuerlinckx, 2007). Furthermore, the data patterns specifically captured by the full DDM are elicited only by certain task designs. In many cases – including many of those where the DDM is used as a measurement model – using the full DDM is asking too much of the data (van Ravenzwaaij, Donkin, & Vandekerckh, 2017).

In cases where it is reasonable to forgo these assumptions of between-trial variability in the parameters of the model, as well as the need for a response bias, parameter estimation is much easier through the ‘EZ’ implementation of the DDM (EZ-diff) introduced by Wagenmakers et al. (2007). EZ-diffwas developed by first constructing a system of equations showing how the DDM parameters can be used to compute a predicted accuracy rate ${{{R}^\text {pr}}}$ and mean ${{{M}^\text {pr}}}$ and variance ${{{V}^\text {pr}}}$ of RTs (the ‘forward’ system).^3^ The insight by Wagenmakers et al. is that the forward system is invertible, so that parameter estimates can be obtained directly from these three summary statistics.

Let $q= \exp \left( -\alpha \nu \right)$. The forward EZ equations are then:

5 $$\begin{aligned} {{{R}^\text {pr}}}= &  \frac{1}{q+ 1} \end{aligned}$$

6 $$\begin{aligned} {{{M}^\text {pr}}}= &  \tau + \left( \frac{\alpha }{2 \nu }\right) \left( \frac{1 - {q}}{1 + {q}}\right) , \end{aligned}$$

7 $$\begin{aligned} {{{V}^\text {pr}}}= &  \left( \frac{\alpha }{2\nu ^3}\right) \left\{ \frac{1-2\alpha \nu {q} - {q}^2}{\left( {q} + 1\right) ^2}\right\} \end{aligned}$$

and parameter estimation is made possible through the following inverse EZ equations:

8 $$\begin{aligned} L= &  \log \left( \frac{{{R}^\text {ob}}}{1-{{R}^\text {ob}}}\right) \nonumber \\ {{{\nu }^\text {es}}}= &  \text {sgn}\!\left( {{R}^\text {ob}}- \frac{1}{2}\right) \sqrt{\frac{L\left( {{R}^\text {ob}}^2 L- {{R}^\text {ob}}L+ {{R}^\text {ob}}- \frac{1}{2}\right) }{{{V}^\text {ob}}}} \end{aligned}$$

9 $$\begin{aligned} {{{\alpha }^\text {es}}}= &  \frac{L}{{{{\nu }^\text {es}}}} \end{aligned}$$

10 $$\begin{aligned} {{{\tau }^\text {es}}}= &  {{M}^\text {ob}}- \left( \frac{{{{\alpha }^\text {es}}}}{2{{{\nu }^\text {es}}}}\right) \left[ \frac{1-\text {exp}\!\left( -{{{\nu }^\text {es}}}{{{\alpha }^\text {es}}}\right) }{1+\text {exp}\!\left( -{{{\nu }^\text {es}}}{{{\alpha }^\text {es}}}\right) }\right] . \end{aligned}$$

Here, ${{{\nu }^\text {es}}}$, ${{{\alpha }^\text {es}}}$, and ${{{\tau }^\text {es}}}$ are parameter estimates, ${{R}^\text {ob}}$ is the observed accuracy rate, ${{M}^\text {ob}}$ is the observed mean of the RTs, and ${{V}^\text {ob}}$ is their observed variance.

## Sometimes EZ-diffusion is the better model

There are use cases in which we believe EZ-diff should be preferred, not because it is easier and faster, but because it is the better model.

EZ-diff gained popularity rapidly as a parameter estimation tool due to its simplicity and practicality. Despite initial criticism calling the EZ implementation “too EZ” – for model fit assessments and for obtaining meaningful parameter interpretations when its assumptions are not met, the trial size is limited, or there are outlier RTs (Ratcliff, 2008) – Wagenmakers, van der Maas, Dolan, and Grasman (2008) showed that EZ-diff can be extended to address most of these cases. Moreover, as van Ravenzwaaij et al. (2017) point out, most binary choice tasks do not elicit the data patterns specifically captured by the full DDM in the first place.

Importantly, van Ravenzwaaij and Oberauer (2009) compared the performance of the full and EZ-diff in a parameter recovery simulation study, and demonstrated that EZ-diff can capture individual differences in true data-generating parameters, while the full model failed to recover individual differences in parameters with across-trial variability. Further, van Ravenzwaaij et al. (2017) used the full DDM to generate data emulating experimental effects and then contrasted the ability of EZ-diff to detect those effects against that of the full model, and were able to conclude that EZ-diff provides a powerful test of simple empirical effects. This advantage was seen again in subsequent validation studies (Arnold, Bröder, & Bayen, 2015) and again in a recent many-modelers study (Dutilh et al., 2019), in which a variation on EZ-diff was the better model in terms of its ability to locate group-level effects. A likely reason for this success is that the mean RT as a summary statistic is less sensitive to random noise in fast RTs, and this robustness is more noticeable in scenarios where the number of trials per participant is low.

Finally, because EZ-diff requires only summary statistics at the individual level, and indeed only requires those summary statistics that are conventionally reported in academic papers, the Bayesian hierarchical extension we will propose next can potentially be applied in a meta-analytic context.

## A probabilistic proxy model for the drift diffusion model

Equations 8-10 provide deterministic estimators of the three diffusion model parameters. To implement EZ-diff in a probabilistic programming language, we require a probabilistic estimator – that is, we need a distribution over data that is conditional on the model parameters. Such a distribution can be derived from Eqs. 5-7 and application of sampling statistics (e.g., Rice, 2006).

If $N$ observations are drawn from a diffusion model whose accuracy rate is ${{{R}^\text {pr}}}$, then the sampling distribution of the observed number of correct trials ${{T}^\text {ob}}$ is:

11 $$\begin{aligned} {{T}^\text {ob}}\sim \text {Binomial}\left( {{{R}^\text {pr}}},N\right) . \end{aligned}$$

Similarly, if $N$ observations are drawn from a sample whose mean and variance of the RTs are ${{{M}^\text {pr}}}$ and ${{{V}^\text {pr}}}$, then the sampling distribution of the observed mean RT ${{M}^\text {ob}}$ is:^4^

12 $$\begin{aligned} {{M}^\text {ob}}\sim \text {Normal}\left( {{{M}^\text {pr}}},\frac{{{{V}^\text {pr}}}}{N}\right) . \end{aligned}$$

Finally, the sampling distribution of the variance of the RTs follows this probability law:^5^

$$\begin{aligned} (N-1)\frac{{{V}^\text {ob}}}{{{{V}^\text {pr}}}}\sim &  \text {Chi-squared}\left( N-1\right) \\ \Rightarrow (N-1)\frac{{{V}^\text {ob}}}{{{{V}^\text {pr}}}}\sim &  \text {Gamma}\left( \frac{N-1}{2},2\right) \\ \Rightarrow {{V}^\text {ob}}\sim &  \text {Gamma}\left( \frac{N-1}{2}, \frac{2{{{V}^\text {pr}}}}{N-1}\right) . \end{aligned}$$

As $N$ becomes sufficiently large, this is well approximated by a normal distribution:^6^

13 $$\begin{aligned} {{V}^\text {ob}}\sim \text {Normal}\left( {{{V}^\text {pr}}}, \frac{2{{{V}^\text {pr}}}^2}{N-1}\right) . \end{aligned}$$

**Fig. 2:**
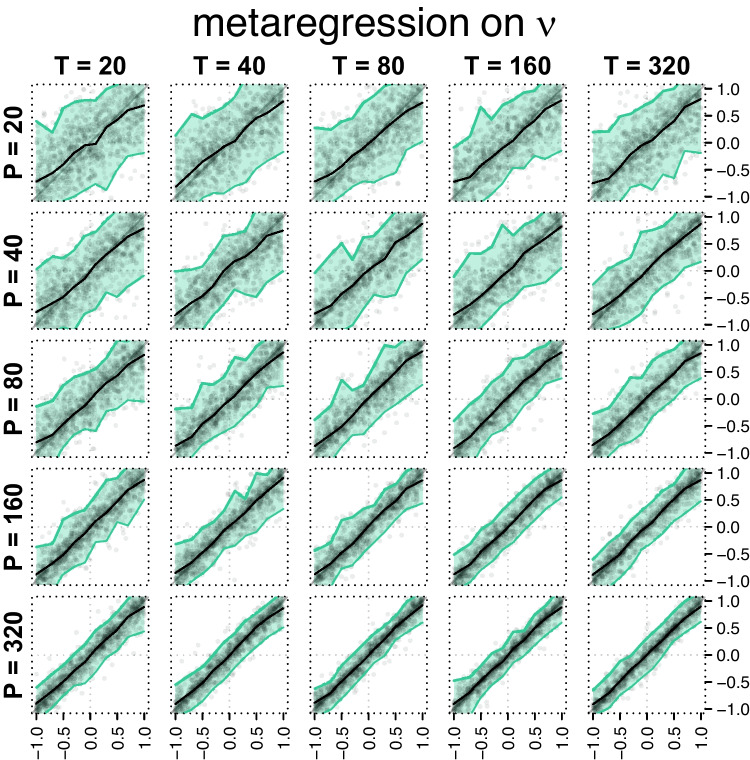
Partial results from the simulation study. Here, we simulated a design in which participants’ individual drift rates were drawn from a normal distribution whose mean was a linear function of some external predictor (as in Eq. 2). Depicted is the recovery of the regression weight $\beta$. We believe that testing a relationship between drift rate and an explanatory covariate would be the typical use of EZ-diff in a cognitive-psychometric context. The 25 panels indicate different combinations of the number of participants *P* and the number of trials *T* per participant. In each panel, the ‘true’ (generated) $\beta$ value is on the horizontal axis and the mean a posteriori estimate is on the vertical axis. The *dark line* in each panel is the median of the estimated $\beta$s. The *shaded area* contains 95% of the estimates (with 2.5% of estimates falling above and below). Recovery is unbiased and the variability decreases rapidly with increasing *P* but only slowly with *T*. This illustrates the benefit of increasing the number of participants over the number of trials (note that the panels on the diagonal from bottom left to top right show scenarios with the exact same total number of observations, but the variability is clearly lower on the left)

Together with Eqs. 5, 6, and 7, Eqs. 11, 12, and 13 provide a predictive distribution of three summary statistics in terms of three DDM parameters – that is, a *likelihood*. We call this set of equations our “proxy model” for the DDM. It is easy to see from Eqs. 11-13 that the EZ-diff estimators in Eqs. 8-10 are the maximum likelihood estimators of the proxy model.

Equipped with a likelihood for EZ-diff, we can now implement it in a probabilistic programming language like JAGS (Plummer, 2003) or Stan (Carpenter et al., 2017) or PyMC (Abril-Pla et al., 2023). This will allow us, among other things, to use this proxy model as a component of a Bayesian hierarchical model.

## The EZ Bayesian hierarchical drift diffusion model

A hierarchical Bayesian extension of EZ-diff allows for a practical and easy implementation of the hierarchical DDM as a measurement model. Equations 1-4 describe the initial hierarchical extension of the DDM (Vandekerckhove et al., 2011). To implement our proxy model, it suffices to substitute the likelihood equation (Eq. 1) with the three equations that constitute our proxy model (Eqs. 11-13). In practical applications, we will also have to adjust data preprocessing code so that mean accuracy and the mean and variance of RTs in each design cell are available. An illustrative implementation of the EZ Bayesian hierarchical drift diffusion model in JAGS is given in Appendix A. The prior distributions in that example can be adjusted for specific applications – ours were inspired by Matzke and Wagenmakers (2009)’s review of the literature.

To test the ability of our proxy model to recover DDM parameters, we conducted a large simulation study. Reproducible code for the simulation study can be found via https://osf.io/bzkpn/ and more extensive detail is in Appendix B. Briefly, we simulated data sets from a hierarchical simple (three-parameter) diffusion model in which *P* participants provide *T* trials in exactly one condition (a between-subjects design). Exactly one of the three DDM parameters was selected as the ‘criterion’ and made a linear function of an exogenous covariate *X* (Eq. 2 shows the case where drift rate was the criterion). *X* was either binary (taking only values of 0 or 1; the “*t* test” design scenario) or took a value between 0 and 1 (inclusive, the “linear regression” design), yielding six distinct scenarios. Additionally, we varied the number of participants and trials per participant, $P \in \{20,40,80,160,320\}$ and $T \in \{20,40,80,160,320\}$, for a total of $6 \times 25 = 150$ conditions. In each condition, we simulated 1000 data sets and used our proxy method (implemented in JAGS using R2jags; Su & Yajima, 2008) to recover the parameters.

Figure 2 shows partial results from this simulation study, and complete results and full details of the implementation are in Appendix B. Across conditions, we observe that our model estimates the beta weights well. Recovery is unbiased even in the case with only 20 participants and improves with increasing *P*. The effect of *T* on recovery is much weaker. Note that the total number of trials in a simulated experiment (across all participants) is the same between each panel and its neighbor to the right and up (or left and down). This way, the figure also illustrates that greater estimation efficiency is often gained by increasing the sample size *P* than the trial count *T*, a known but underappreciated feature of hierarchical models (Cohen, Sanborn, & Shiffrin, 2008; DeKay, Rubinchik, Li, & De Boeck, 2022; Rubinchik, 2019; Vandekerckhove, 2024).

We additionally evaluate the recovery of the population means of the model parameters. The mean drift rate is generally recovered well with no notable bias. The mean boundary separation is recovered well, with a slight bias towards the mean of the prior in the case where we are recovering $\mu _\alpha$ as the *intercept* of a regression with $P = 20$. The mean nondecision time shows the same pull towards the mean of the prior even with medium values of *P*. Moreover, $\mu _\tau$ is frequently systematically underestimated even with large *P* and *T*. Note, however, that the fact that this bias is *systematic* means that the usefulness of the model is preserved – the effect of a covariate predictor is still estimated well.

In summary, the simulation study illustrates good recovery properties – especially of the beta weight parameter – but also shows that our proxy model inherits the estimation biases from EZ-diff. Consequently, we recommend the use of the EZ Bayesian hierarchical drift diffusion model specifically in the cognitive-psychometrical context – that is, scenarios where the interest is not in specific values of DDM parameters, but rather in *regression coefficients that link DDM parameters to external covariates* such as explanatory predictors or elements of an experimental design (i.e., differences between conditions).

## Example applications and additional illustrations

To further illustrate the usefulness of our proxy model, we provide a number of worked-out applications. These applications can be found as appendices.

In Appendix C, we conduct a short simulation study with a within-subjects *t* test design and illustrate an easy-to-use hypothesis testing procedure with a Bayes factor (Kass & Raftery, 1995). The Bayes factor has excellent inferential properties even for the case with only 20 participants with 20 observations.

In Appendix D, we revisit data from Ratcliff and Rouder (1998) and illustrate a more complex regression case. Despite the complexity, the code is simple enough that it can be implemented in the Bayesian analysis package JASP (Love et al., 2015).

Finally, in Appendix E, we revisit data from Vandekerckhove, Panis, and Wagemans (2007) and illustrate a practical hypothesis testing scenario in an incomplete ANOVA design.

## Summary

The drift diffusion model (DDM) is a popular model of choice response time that comes at a significant computational cost. Moreover, many of its applications add computational complexity above and beyond that of calculating the expensive DDM likelihood. Such costly applications have included Bayesian hierarchical extensions (Vandekerckhove et al., 2011), latent variable structures (Vandekerckhove, 2014), applications to large population samples (Lucio et al., 2017; Salum et al., 2014a, b), use of DDMs as a component in numerical experiments (Stafford, Pirrone, Croucher, & Krystalli, 2020), real-time adaptive design optimization (Bahg et al., 2020), and model comparisons that involve high-dimensional numerical integration (Boehm et al., 2023; Gronau, Heathcote, & Matzke, 2019).

The EZ-diffusion model (EZ-diff; Wagenmakers et al., 2007) was a significant development in this regard, allowing for rapid approximations to DDM parameters with relative ease. However, enhancing EZ-diff with additional statistical structure – such as constraints on parameters over conditions, or introduction of explanatory covariates – was not yet possible. We have introduced the EZ Bayesian hierarchical DDM, a new formulation of EZ-diff as a Bayesian generative model that lends itself to implementation in generic probabilistic programming languages. The derivation was based in standard mathematical statistics – the key equations are Eqs. 11, 12, and 13. We conducted simulation studies showing good recovery of DDM parameters using our new method, including good recovery of hierarchical regression parameters, and we provide some examples in an online repository (https://osf.io/bzkpn/).

With this new ‘EZ’ formulation, using the DDM is no more costly than using a normal distribution, opening the door to a wide array of modeling applications.

## Figures and Tables

**Fig. 3:**
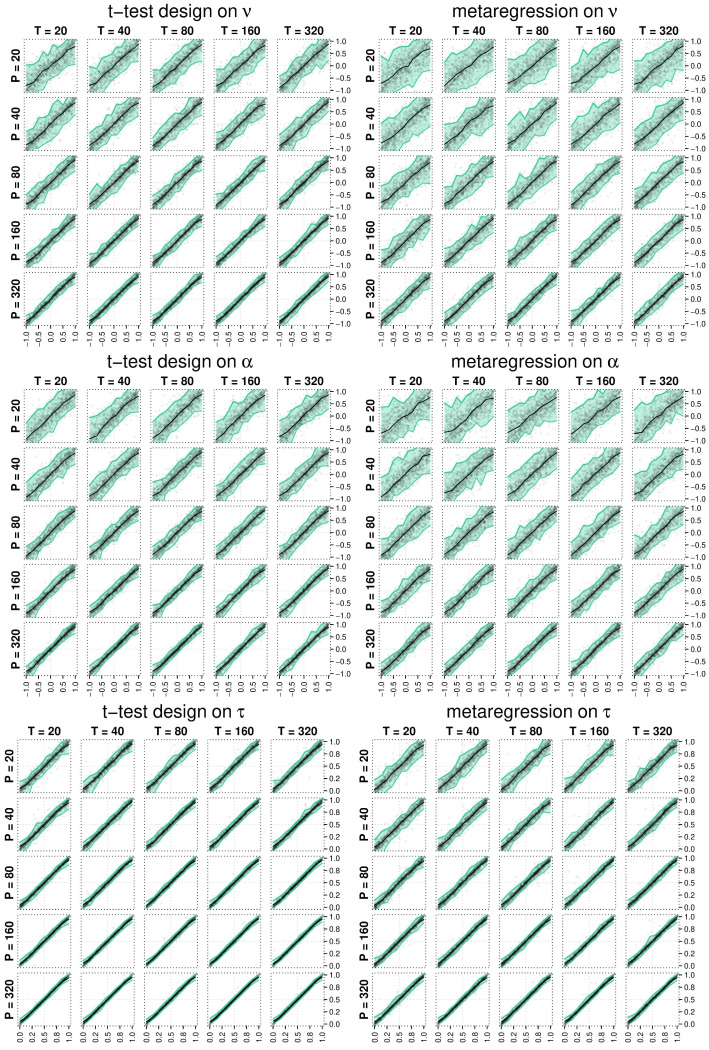
Recovery of the $\beta$ parameter across simulation studies (true values on the horizontal axis). The *left three panels* show studies with ‘*t* test’ designs, and the *right panels* show ‘linear regression’ designs. *Rows* correspond to different criterion parameters: drift rate ($\nu$, *top row*), boundary separation ($\alpha$, *middle row*), and nondecision time ($\tau$, *bottom*). *Shaded areas* indicate where $95\%$ of the estimated values fell, and the *thick black lines* represent median recovered values

**Fig. 4:**
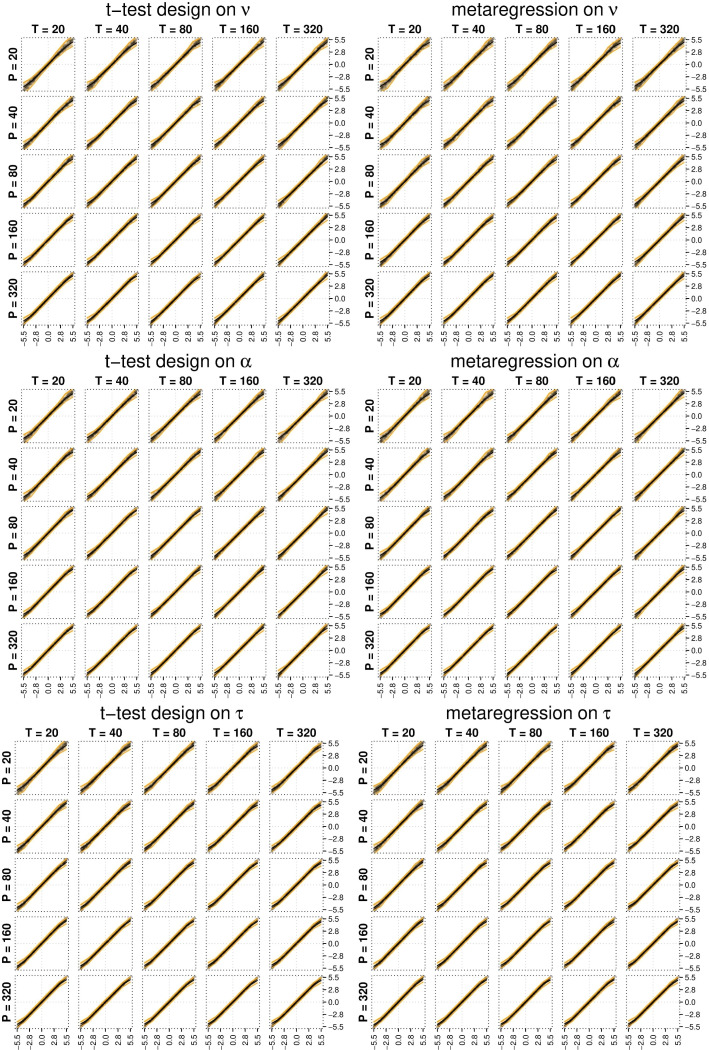
Recovery of the population mean drift rate $\mu _\nu$ parameter across simulation studies (true values on the horizontal axis). The *left three panels* show studies with ’*t* test’ designs, and the *right panels* show ’linear regression’ designs. *Rows* correspond to different criterion parameters: drift rate ($\nu$, *top row*), boundary separation ($\alpha$, *middle row*), and nondecision time ($\tau$, *bottom*). *Shaded areas* indicate where $95\%$ of the estimated values fell, and the *thick black lines* represent median recovered values

**Fig. 5:**
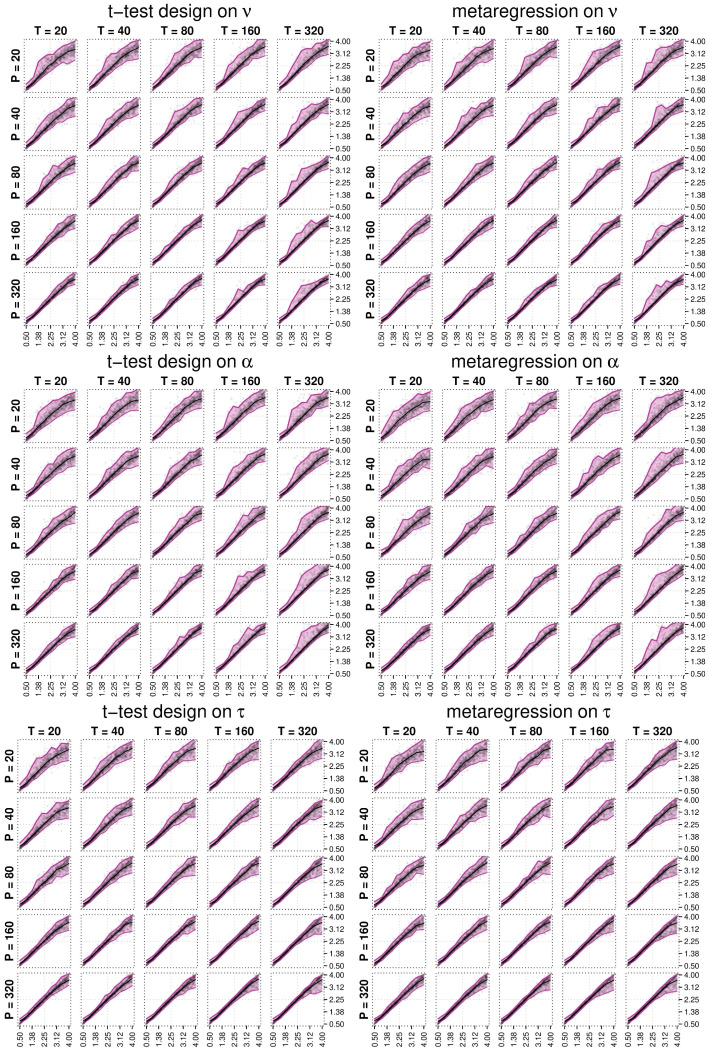
Recovery of the population mean boundary separation $\mu _\alpha$ parameter across simulation studies (true values on the horizontal axis). The *left three panels* show studies with ‘*t* test’ designs, and the *right panels* show ‘linear regression’ designs. *Rows* correspond to different criterion parameters: drift rate ($\nu$, *top row*), boundary separation ($\alpha$, *middle row*), and nondecision time ($\tau$, *bottom*). *Shaded areas* indicate where $95\%$ of the estimated values fell, and the *thick black lines* represent median recovered values

**Fig. 6:**
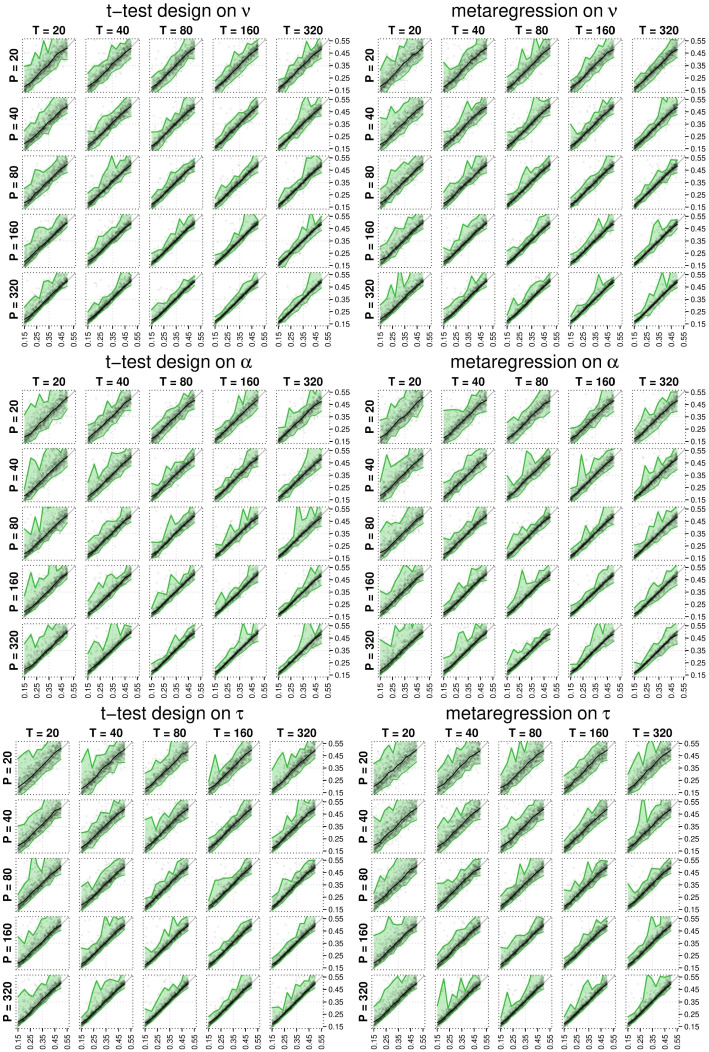
Recovery of the population mean nondecision time $\mu _\tau$ parameter across simulation studies (true values on the horizontal axis). The *left three panels* show studies with ‘*t* test’ designs, and the *right panels* show ‘linear regression’ designs. *Rows* correspond to different criterion parameters: drift rate ($\nu$, *top row*), boundary separation ($\alpha$, *middle row*), and nondecision time ($\tau$, *bottom*). *Shaded areas* indicate where $95\%$ of the estimated values fell, and the *thick black lines* represent median recovered values

**Fig. 7:**
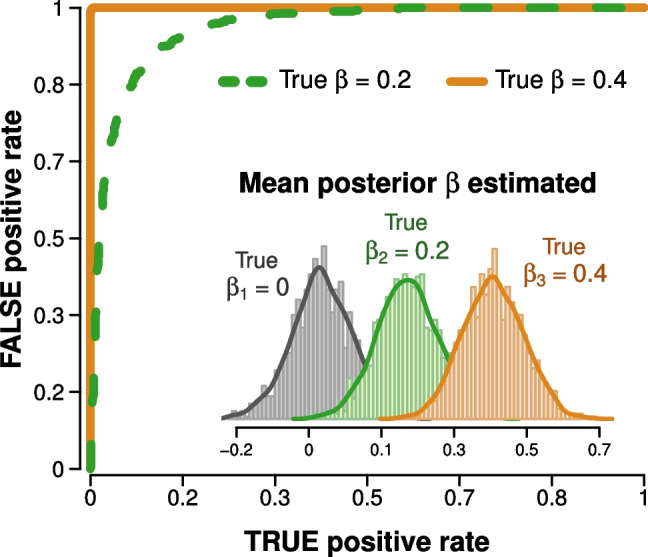
*Main panel:* Receiver operating characteristic curves illustrating the trade-off between true positive rate (*vertical axis*) and false-positive rate (*horizontal axis*) for different Bayes factor thresholds (*solid line*: true $\beta =0.4$; *dashed line*: true $\beta =0.2$). *Inset:* Distributions of estimated $\beta$ parameters for each condition

**Fig. 8:**
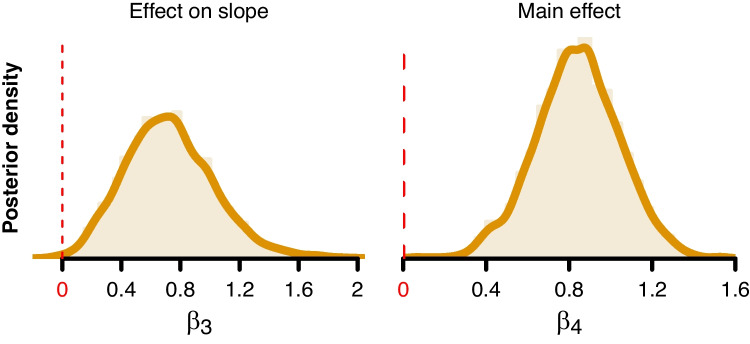
Posterior distributions for the effect on slope ($\beta _3$, *left panel*) and the main effect ($\beta _4$, *right panel*) of the instruction condition on the drift rate. The *red vertical lines* indicate the ’no-effect’ values $\beta _3 = 0$ and $\beta _4 = 0$

**Fig. 9:**
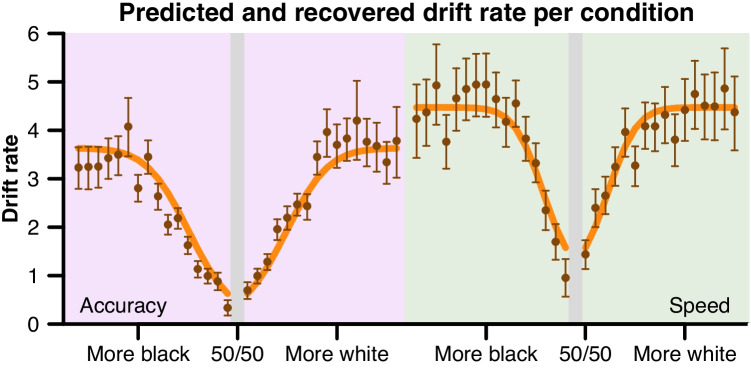
Predicted $\nu ^\text {pred}_{i,s}$ and estimated $\nu _{i,s}$ drift rates across conditions. The *thick orange lines* show the posterior mean predicted drift rates $\nu ^\text {pred}_{i,s}$ for every stimulus configuration level across instruction conditions. The *brown dots* correspond to the posterior mean drift rates estimated $\nu _{i,s}$, with the *brown whiskers* indicating their corresponding $95\%$ CI. The ‘Accuracy’ and ‘Speed’ instruction conditions are shown on the left and right panels, respectively. The *vertical gray bars* indicate the 50/50 black-and-white condition omitted from our analysis for lack of a ‘correct’ response

**Fig. 10:**
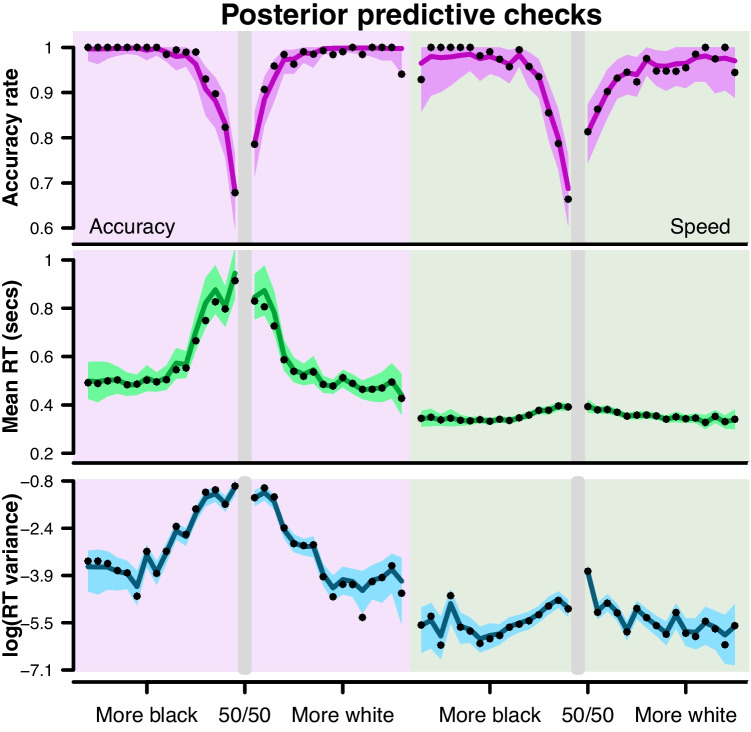
Posterior predictive checks for all EZ summary statistics: the accuracy rate (*top panel*), the mean RT in seconds (*middle panel*), and the RT variance expressed in a log scale (*bottom panel*). All panels are split by instruction condition, with the ‘Accuracy’ condition on the left and the ‘Speed’ condition on the right. *Thick colored lines* show the mean posterior predictions for each stimulus configuration level and instruction condition, with the *shaded area* marking the $95\%$ CI. The *black markers* indicate the actual summary statistics computed from the data analyzed

**Fig. 11:**
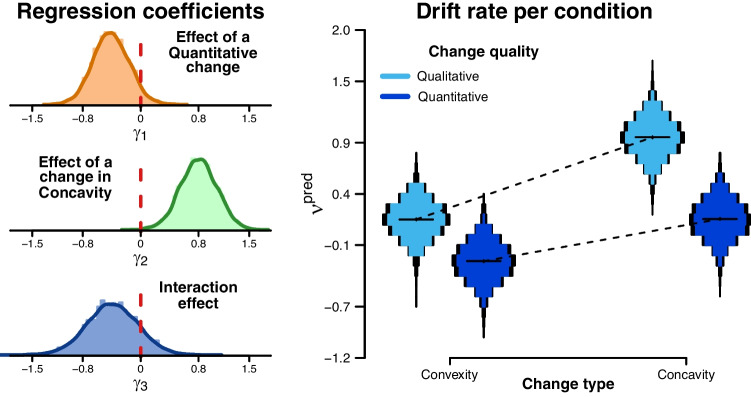
Posterior distributions of the regression coefficients $\gamma _1$, $\gamma _2$ and $\gamma _3$, and posterior distributions of the predicted drift rates across all possible combinations of change type $\times$ change quality. The *top left panel* shows the posterior distribution of the main effect of the change quality $\gamma _1$. The *top right panel* presents the posterior distribution of the main effect of change type $\gamma _2$. The *bottom left panel* displays the posterior distribution of the interaction effect between change type and change quality. All these distributions mark the zero-value position with a *vertical dashed red line*. The *bottom right panel* presents the posterior distributions for the predicted drift rates across all change type $\times$ change quality combinations

## Data Availability

Not applicable.

## Appendix A: Illustrative JAGS code

The following JAGS code implements the EZ Bayesian hierarchical drift diffusion model, with drift rate as the criterion of a regression with predictor *X*. The code consists of a block of priors, a block for the hierarchical level with the regression component, a block with the forward EZ equations, and a block with the sampling distributions for each summary statistic. Note that JAGS uses precision (inverse variance) as the second parameter of dnorm().

The code is written to expect a data set with five variables, all vectors of length equal to the number of cells in the study design (here, there is one cell per participant because the design is fully between participants):

- nTrials: the total number of trials in this cell
- correct: the number of correct responses
- meanRT: the mean of the RTs (in seconds)
- varRT: the variance of the RTs (in seconds squared)
- X: the predictor

In the remaining appendices, we provide more complex examples. All of our code, including applications imported into JASP (Love et al., 2015), can be accessed via https://osf.io/jstgw/.

## Appendix B: Parameter recovery simulation studies

We present the results of six simulation studies to test the ability of our proxy model to recover the parameters of the hierarchical DDM. In all studies, we simulated two-alternative forced-choice data from *P* participants $P \in \{20,40,80,160,320\}$, each providing *T* trials $T \in \{20,40,80,160,320\}$. In all cases reported below, we simulated a between-subjects design.

### Sampling procedure

All simulated participants had a randomly chosen drift rate $\nu _p$, boundary separation $\alpha _p$, and nondecision time $\tau _p$. The population-level distributions of all parameters were normal (see Eqs. 2-4). In all simulations, we chose one parameter $\theta \in \left\{ \nu , \alpha , \tau \right\}$ as the ‘criterion’ and made its population mean a linear function of a single person-level predictor $x_p$:

$$\theta _p \sim N(\mu _\theta + \beta x_p,\sigma ^2_\theta ).$$

Here, the beta weight $\beta$ is the parameter of greatest interest, as it describes the prediction of $\theta$ by $x_p$ (i.e., it is an external covariate).

In the six simulation studies we describe below, $\theta$ was one of $\nu$, $\alpha$, or $\tau$, and $x_p$ was either binary (the “*t* test” design scenario) or took a value between 0 and 1 (inclusive; the “linear regression” design). Every study consisted of 1000 iterations in which a data set was generated from these individual-level sampled parameter values.

Each iteration of each simulation study began by drawing a random set of hierarchical mean parameters:

$$\begin{aligned} \mu _\alpha\sim &  U( 0.50,4.00)\\ \mu _\nu\sim &  U(-5.50,5.50)\\ \mu _\tau\sim &  U( 0.15,0.50) \end{aligned}$$

A set of hierarchical standard deviation parameters $\sigma _\alpha$, $\sigma _\nu$ and $\sigma _\tau$ was then defined to be proportionate to the hierarchical mean parameters sampled. This manipulation served the purpose of ensuring lower-value hierarchical means were not matched with higher-value standard deviations.

The beta weight parameter $\beta$ to be used in any iteration was also sampled from a uniform distribution. For simulations with $\alpha$ or $\nu$ as the criterion, the range was $(-1,1)$. When $\tau$ was the criterion, $\beta$ was sampled from a (0, 1) uniform interval.

We then drew *P* person-specific diffusion model parameters from the population-level distributions defined by these hierarchical parameters. Finally, for each person, we drew *T* realizations of the diffusion process using the rejection-based algorithm described in Tuerlinckx, Maris, Ratcliff, and De Boeck (2001). EZ-diff was developed from the simple DDM with no initial bias and no between-trial variability parameters. As such, the data simulated for every participant did not include any form of between-trial variability.

### Parameter recovery

For parameter recovery, we drew samples from the posterior distribution of each of the hierarchical parameters using the JAGS code in Appendix A (with the regression structure applied to whichever parameter was the criterion in that simulation). For each simulation study, we drew three MCMC chains with 2500 samples after a 500-sample burn-in period.

### Speed

The posterior sampling, together with standard postprocessing in R, generally took between four and five seconds per iteration on an off-the-shelf desktop computer.

### Evaluation

We evaluate the recovery by plotting true values on the horizontal axis and mean a posteriori estimates on the vertical axis in Figs. 3 (for $\beta$), 4 (for $\mu _\nu$), 5 (for $\mu _\alpha$), and 6 (for $\mu _\tau$). Each figure contains six panels of 25 (5$\times$5) scatter plots. The panels in the left column refer to the “*t* test” designs and those in the right column to the “linear regression” designs. The three rows of panels indicate the cases with the drift rate $\nu$ as criterion (top row), the boundary separation $\alpha$ as criterion (middle row), and the nondecision time $\tau$ as criterion (bottom row). Better recovery is signaled by points falling closer to the gray dashed diagonal. The shaded areas denote 95% of the estimates obtained across different regions of the true parameter space (2.5% falling on either side), with the thick black lines indicating the median estimates.

Note that Figs. 4, 5, and 6 show the recovery of parameter-specific hierarchical means ($\mu _\nu$, $\mu _\alpha$, and $\mu _\tau$, respectively) and that the role of these parameters depends on the design. That is, in Fig. 4, the panels on the top row (“*t* test on $\nu$” and “metaregression on $\nu$”) show the recovery of $\mu _\nu$, which in these cases is the intercept of the linear function $\mu _\nu +\beta x_p$. The same is true for the panels in the middle row in Fig. 5 and the bottom row in Fig. 6.

The 25 scatter plots differ in the number of simulated participants *P* and trials per participant *T*. As expected, the precision of recovery improves as *P* and *T* increase. The values of *P* and *T* were chosen so that the total number of observations is equal between each scatter plot and its neighbor to the top right or bottom left. This helps illustrate that recovery of the beta weights improves faster with *P* than with *T* (Cohen et al., 2008). For small *P* and *T* we sometimes see a strong effect of the prior (i.e., extreme values are pulled towards the middle), and for mean nondecision time we see occasional large overestimates.

In summary, the simulation studies illustrate good recovery properties of the mean drift rate parameter and the beta weight parameter, but also show that our proxy model inherits the estimation biases from EZ-diff.

## Appendix C: A brief simulation exercise on hypothesis testing

We conducted a simulation study with a within-subjects *t* test design and two experimental conditions. Data was generated according to the following hierarchical structure:

$$\begin{aligned} \nu _{p,k}\sim &  \text {Normal}\left( \mu _\nu + \beta X_{k}, 0.25\right) \nonumber \\ \alpha _{p}\sim &  \text {Normal}\left( \mu _\alpha , \sigma _\alpha \right) \nonumber \\ \tau _{p}\sim &  \text {Normal}\left( \mu _\tau ,\sigma _\tau \right) \nonumber \\ \textbf{y}_{p,k,i}\sim &  \text {Wiener}\left( \alpha _i, \tau _i, \nu _{i,k}\right) , \end{aligned}$$

so that choice and RT data $\textbf{y}_{p,k,i}$ from trial *i* of participant *p* in condition $k \in \{1,2\}$ was simulated from a simple diffusion process with participant-specific boundary separation $\alpha _p$ and nondecision time $\tau _p$ parameters, and participant-by-condition specific drift rates $\nu _{i,k}$. We used the indicator variable $X_k: \{ X_1 = 0, X_2 = 1 \}$ to enforce a *t* test design on the drift parameter and a beta weight $\beta$ parameter to capture the within-participant differences in performance across the two conditions.

We generated 1000 data sets with 20 participants and 20 trials per condition across three fixed effects conditions: $\beta \in \{0.0,0.2,0.4\}$. Each iteration of the simulation study began by sampling hierarchical parameters from suited uniform distributions. From there, participant and participant-by-condition specific parameters were generated to simulate the data.

### Estimation

Once the trial-by-trial data had been generated, we computed the EZ-diff summary statistics for each participant-by-condition design cell. Estimation was conducted using the JAGS model below. We tested convergence of the MCMC algorithm by confirming that the potential scale reduction factor $\hat{R}$ was less than 1.05. If the algorithm failed to converge (approx. 0.01% of iterations), the iteration was discarded.

### Speed

When fitting the JAGS model, we drew two MCMC chains with 1500 samples after a 500-sample burn-in period. The posterior sampling and standard postprocessing in R took approximately five seconds per iteration on an off-the-shelf desktop computer.

### Hypothesis testing procedure

The goal of this short simulation study is to demonstrate a simple Bayesian hypothesis testing procedure. We are interested in testing whether the simulated experimental condition had an effect on the population mean drift rate. For each iteration in our simulation study, we compute a Bayes factor (Kass & Raftery, 1995) to evaluate the constraint that $\beta \approx 0$. The Bayes factor favoring the alternative is simply the ratio between the prior mass near 0 and the posterior mass near zero:

$$ B = \frac{P_\text {prior}\left( \beta \in \left( -\varepsilon ,\varepsilon \right) \right) }{P_\text {posterior}\left( \beta \in \left( -\varepsilon ,\varepsilon \right) \right) }, $$

for some small $\varepsilon$ (we used 0.1). We then decide that $\beta$ is nonzero if *B* exceeds some threshold that is determined by the strength of evidence we need. In Fig. 7, we show the resulting receiver operating characteristic (ROC) curve. Even with this small data size, the ROC curve demonstrates excellent sensitivity and selectivity for $\beta$.

## Appendix D: Applied example: Metaregression

In this example, we showcase the advantages of implementing our proxy model with a meta-regression extension that involves a non-linear function. We re-analyze data from Ratcliff and Rouder (1998), who conducted a numerosity study in which participants had to identify the brightness of pixel arrays shown on screen as “high” or “low.” The pixel arrays varied across 33 configuration levels that differed in the proportion of white pixels, with the first 16 levels indicating a majority of black pixels. The task incorporated two instruction conditions that primed participants to prioritize the speed or the accuracy of their responses. We analyze the data from a single participant, excluding the stimulus configuration with an equal number of black and white pixels (for which there is no correct response).

### The model

The model has two components. The main component is the proxy model built from the sampling distributions for the summary statistics computed from the data. The second component is a meta-regression structure with which we incorporate an effect $\beta$ of instruction $X_i$ on the boundary separation $\alpha$,

$$ \alpha _i \sim \text{ Normal }(\mu _\alpha +\beta X_i,\sigma _\alpha ). $$

and a nonlinear regression on the drift rate $\nu$ using instruction $X_i$ and stimulus configuration $Z_s$ as predictors:

$$\begin{aligned} S_{i,s}= &  \Phi (\beta _1+\beta _2 |Z_s|+\beta _3X_i|Z_s|) \\ \nu ^\text {pred}_{i,s}= &  \mu _\nu +\beta _0 S_{i,s}+\beta _4 X_i \\ \nu _{i,s}\sim &  \text{ Normal }(\nu ^\text {pred}_{i,s},\sigma _\delta ). \end{aligned}$$

Here, $Z_s$ is the difference between the number of black and white pixels (standardized). We use the absolute value $|Z_s|$ since we expect task difficulty to change as the proportion of white pixels departs from $50\%$, regardless of the direction of the difference.

### Results

We use the proposed metaregression structure to explore the effect of instruction on the drift rate. Figure 8 presents the posterior distributions for the regression coefficients of interest: the effect on slope $\beta _3$ and the main effect $\beta _4$ of instruction on the drift rate parameter. Both panels show very low posterior density near zero, suggesting that the instruction condition had an effect on the drift rate parameter.

Figure 9 shows the posterior mean predicted drift rates $\nu ^\text {pred}_{i,s}$ (thick, orange line) and the posterior mean estimated drift rates $\nu _{i,s}$ (brown markers) with their respective 95$\%$ CI (brown whiskers) for each stimulus configuration level *s* and instruction condition *i*. As expected, drift rates decrease in both instruction conditions as the stimulus configuration approaches the 50/50 condition, where the task is more difficult. Moreover, as suggested by Fig. 8, drift rates seem to be shifted upwards in the ‘Speed’ instruction condition, indicating an effect of instruction condition on drift rate.

Finally, to demonstrate the adequacy of our meta-regression model, in Fig. 10 we present the posterior predictive checks obtained with respect to all three EZ summary statistics. In all panels, the thick line and shaded region indicate the mean posterior prediction and 95$\%$ CI, respectively, with the black dots corresponding to the summary statistics computed from the data for each stimulus configuration and instruction condition. Overall, our model provides an adequate account of the data analyzed.

### Speed

This analysis with 7802 observations after data cleaning took 8.6 s on an off-the-shelf desktop computer.

## Appendix E: Applied example: Hypothesis testing

This example features an implementation of our proxy model with a meta-regression extension that uses a multiple linear regression function for hypothesis testing. We re-analyze the data from nine participants in a shape perception study (Vandekerckhove et al., 2007), who were asked to compare pairs of irregular shapes in a “Same/Different” task. The experimental design included three factors: (1) whether there was a change between the shapes, and if so (2) what was the change type (did it affect a *convexity* or *concavity*); and (3) the change quality (did it introduce a *qualitatively* new vertex or did it *quantitatively* change an existing one). Due to the incompletely crossed design, this leads to five experimental conditions (see Table 1).

### The model

We explore the variability in the drift rate parameter $\nu _k$ across conditions *k* with the following multiple linear regression model:

$$\begin{aligned} \nu ^\text {pred}_k&= \mu +A_k(\gamma _1B_k+\gamma _2C_k+\gamma _3B_kC_k)+(1-A_k)\gamma _4\\ \nu _k&\sim \text{ Normal }(\nu ^\text {pred}_k,\sigma _\nu ). \end{aligned}$$

**Table 1:** Experimental conditions in Vandekerckhove et al. (2007)

Condition	Change (*A*)	Change quality (*B*)	Change type (*C*)
$k = 1$	Yes ($A = 1$)	Qualitative ($B = 0$)	Convexity ($C = 0$)
$k = 2$	Yes ($A = 1$)	Quantitative ($B = 1$)	Convexity ($C = 0$)
$k = 3$	Yes ($A = 1$)	Qualitative ($B = 0$)	Concavity ($C = 1$)
$k = 4$	Yes ($A = 1$)	Quantitative ($B = 1$)	Concavity ($C = 1$)
$k = 5$	No ($A = 0$)	n/a	n/a

**Table 2:** Regression coefficients for the predicted drift rates

K	Drift predicted ($\nu _{\text {pred},k}$)	Interpretation of parameter added
1	$\mu _\nu$	$\mu _\nu$ is the baseline drift rate
2	$\mu _\nu$ + $\gamma _1$	$\gamma _1$ is the effect of a quantitative change
3	$\mu _\nu$ + $\gamma _2$	$\gamma _2$ is the effect of a change in concavity
4	$\mu _\nu$ + $\gamma _1$ + $\gamma _2$ + $\gamma _3$	$\gamma _3$ is an interaction term
5	$\mu _\nu$ + $\gamma _4$	$\gamma _4$ is the effect of not having any change

The predicted drift rate $\nu ^\text {pred}_k$ for each condition *k* is determined by its unique configuration of the dummy variables *A*, *B*, and *C* (see Table 1). Table 2 summarizes the drift rates predicted per condition and the implied interpretation for each model parameter. We will focus on the regression effects $\gamma _1$, $\gamma _2$, and $\gamma _3$.

### Results

We use the multiple linear regression model shown above to explore the effect of change quality and change type on the drift rate parameter. The top two panels in Fig. 11 show the posterior distributions for the main effects of change quality $\gamma _1$ and change type $\gamma _2$. These posterior distributions assign a low – but not nonzero – posterior density around zero (marked with a red dashed vertical line). To evaluate the strength of evidence these estimates provide, we can compute Bayes factors against a restricted model in which the weight is close to 0 (as in Appendix C, we use a tolerance of 0.1, meaning that we test the notion that the weight is less than 0.1 in absolute value). For $\gamma _1$, we obtain a Bayes factor of 0.93, which is close enough to 1 as to be nearly perfectly equivocal. For $\gamma _2$, we obtain a Bayes factor of 26, which is strong evidence for a difference between the concavity and convexity conditions.

The bottom left panel in Fig. 11 shows the posterior distribution for the interaction effect of change type and change quality $\gamma _3$, which has some posterior density around zero despite being centered around $-0.45$. This suggests that there is at best weak evidence for an interaction effect between these two factors. Indeed, when we compute the Bayes factor against $\gamma _3 \in (-0.1,0.1)$, we get a value of 0.67, again close to 1.

In the bottom right panel of Fig. 11, we show the posterior distributions of the predicted drift rates across each possible combination of change quality and change type. We see a clear effect of change type (concavity vs. convexity), but the other effects are more murky.

### Speed

Fitting the JAGS model for this example on the 5,722 observations after data cleaning took 3.8 seconds in an off-the-shelf desktop computer.

## Footnotes

1 Indirect inference has recently come to imply the use of simulated data sets to fit models and estimate parameters, but that is not what we will do here.

2 Although care should be taken with parameters that are naturally bounded to be positive. Sometimes it is preferable to apply a linear regression to $\log \!\left( \alpha \right)$ or $\log \!\left( \tau \right)$ instead.

3 In the original formulation of EZ-diff, a distinction is made between the distribution of ‘correct’ versus ‘incorrect’ RTs. However, under the drift diffusion model with only three parameters, the mean and variance of the decision time do not depend on the response (Stone, 1960), so we can let go of this distinction.

4 Here the normal distribution is expressed with a mean and variance parameter.

5 Here the gamma distribution is expressed with a shape and scale parameter.

6 This approximation is not strictly speaking necessary since we could implement the gamma distribution directly. However, the normal distribution affords more flexibility for model-building, and the gamma is not as numerically stable.

## Code Availability

Our simulation studies can be reproduced using code available via GitHub, https://github.com/joachimvandekerckhove/ezbhddm (Python) and https://github.com/Adrifelcha/EZ-project (R), and archived on the Open Science Framework (https://osf.io/bzkpn/). The Python codebase is in the src/ directory and R/ contains R code. Python and R notebooks to repeat small-scale simulations and create figures are in the notebooks/ directory. Finally, the vm/ directory contains set-up information for a virtual machine with a suitable computational environment for reproducibility. Code and example applications are additionally provided via https://osf.io/bzkpn/.
